# Diphtheria—Phytolacca

**Published:** 1884-12

**Authors:** Walter M. James

**Affiliations:** Philadelphia


### DIPHTHERIA—PHYTOLACCA.

WALTER M. JAMES, M. D., PHILADELPHIA.

In May last I was called to see a child suffering from sore throat. Upon inspection the appearances strongly suggested diphtheria. Being quite uncertain of the diagnosis, as well as the indicated remedy, I gave Sac. lac. and decided to wait until the morrow for further developments.

The next day the diagnosis was confirmed ; it was a case of diphtheria of a most malignant type. The tongue was broad and flabby, the edges quite red, with ulcers here and there upon it, the saliva abundant and having a most fetid odor. The tonsils were much swollen, and in a few hours later the submaxillary glands became enormously enlarged. The child showed a strong tendency to stupor.

My first thought was of Mercury. But there was an absence of the indentations upon the edge of the tongue and the profuse sweat. Baptisia was finally given. The next morning the stupor had almost disappeared. Improvement continued thus from day to day for a week very slowly, sometimes seeming to be suspended yet the patient never retrograding. Finally vomiting set in. Instead of giving a remedy hap-hazard, I tried to find reliable indications.

The principal symptoms presenting indications were the following :

1. Profuse saliva.
2. Ulceration of tonsils.
3. A membrane hanging from the palate down the throat like a curtain.
4. Vomiting.

All these symptoms appeared under Phytolacca in *Hering's Condensed Materia Medica.*

Accordingly, I gave this remedy. The vomiting stopped at once. The membrane fell off in twenty-four hours and was expectorated, and the boy made a good recovery.

